# Preparation and Characterization of Porous Cellulose Acetate Nanofiber Hydrogels

**DOI:** 10.3390/gels9060484

**Published:** 2023-06-13

**Authors:** Lijie Jiang, Xingyu Huang, Chaochao Tian, Yidan Zhong, Ming Yan, Chen Miao, Ting Wu, Xiaofan Zhou

**Affiliations:** 1 National-Provincial Joint Engineering Research Center of Electromechanical Product Packaging, Nanjing Forestry University, Nanjing 210037, China; 2 Jiangsu Provincial Key Lab of Pulp and Paper Science and Technology, College of Light Industry and Food, Nanjing Forestry University, Nanjing 210037, China; 3 Key Laboratory of Recycling and Eco-Treatment of Waste Biomass of Zhejiang Province, Zhejiang University of Science and Technology, Hangzhou 310023, China; 4 College of Bioresources Chemical and Materials Engineering, Shaanxi University of Science and Technology, Xi’an 710021, China; 5 Institute of Chemical Industry of Forest Products, Chinese Academy of Forestry, Key Lab of Biomass Energy and Material of Jiangsu Province, Nanjing 210042, China

**Keywords:** cellulose acetate, hydrogel, porous, nanofiber

## Abstract

The currently reported methods for preparing cellulose acetate hydrogels use chemical reagents as cross-linking agents, and the prepared ones are non-porous structured cellulose acetate hydrogels. Nonporous cellulose acetate hydrogels limit the range of applications, such as limiting cell attachment and nutrient delivery in tissue engineering. This research creatively proposed a facile method to prepare cellulose acetate hydrogels with porous structures. Water was added to the cellulose acetate–acetone solution as an anti-solvent to induce the phase separation of the cellulose acetate–acetone solution to obtain a physical gel with a network structure, where the cellulose acetate molecules undergo re-arrangement during the replacement of acetone by water to obtain hydrogels. The SEM and BET test results showed that the hydrogels are relatively porous. The maximum pore size of the cellulose acetate hydrogel is 380 nm, and the specific surface area reaches 62 m^2^/g. The porosity of the hydrogel is significantly higher than that of the cellulose acetate hydrogel reported in the previous literature. The XRD results show that the nanofibrous morphology of cellulose acetate hydrogels is caused by the deacetylation reaction of cellulose acetate.

## 1. Introduction

In recent years, hydrogels have attracted wide attention in the biological field. Hydrogels are hydrophilic polymer networks which may absorb from 10–20% (an arbitrary lower limit) up to thousands of times their dry weight in water [1,2]. Many unique properties make hydrogels attractive biomaterials, such as high porosity, biocompatibility, bionic properties, and similar physical properties of natural tissue. These properties result in hydrogels being widely studied in the biological field, including in drug delivery systems, tissue engineering scaffolds, and biomedical devices [3,4].

Hydrogels can be classified as natural or synthetic hydrogels, depending on the hydrogel raw material [5]. Because of the increasing focus on environmental problems associated with synthetic polymers, there is an emerging tendency toward the use of naturally occurring polymers instead of synthetic ones [6]. Natural polymer hydrogels are excellent candidates for biological applications due to their biodegradability, biocompatibility, and low stimulation of the host immune response. Various natural polymers, such as gelatin [7], cellulose, and their derivatives [8,9], have been used to fabricate biopolymer-based hydrogels. Among the biopolymers, cellulose is the most abundant natural polymer in nature and can be considered an inexhaustible resource [10], which has the advantages of non-toxicity, biocompatibility, and biodegradability [11]. However, cellulose is insoluble in common organic solvents due to its inherent crystalline structure [4,10]. In addition, cellulose-based materials still face the problem that in vivo degradability is limited but degradability can be induced by modifications that disturb its higher-ordered structure [12,13,14]. Therefore, cellulose is often derivatized to facilitate its solubilization and processing.

Cellulose acetate is one of the derivatives of cellulose, which is produced by replacing the hydroxyl group in cellulose disaccharides with acetyl groups [15,16], and has the advantages of abundant source and sustainability [13]. Cellulose acetate has a lower crystallinity than that cellulose, and therefore, cellulose acetate has a higher solubility in common solvents [17]. Cellulose acetate with a (degree of substitution) DS < 1 is soluble in aqueous solutions, whereas those with a DS > 1 tend to be insoluble in aqueous solutions, but soluble in many organic solvents, which facilitates the large-scale application of cellulose acetate. Cellulose acetate has been used in cell culture [18], skin tissue engineering [12], and drug release [19], as an adhesive and as a component of pharmaceuticals. Cellulose acetate also has good mechanical properties (the elastic modulus is 4.69–4.91 GPa) [20], biocompatibility, and affinity for various biological substances.

Meanwhile, cellulose acetate is a good material for the preparation of hydrogels. Recent studies have shown that cellulose acetate can be synthesized into hydrogels through chemical cross-linking reactions. Senna et al. [6] prepared cellulose acetate hydrogels by dissolving cellulose acetate with dimethylformamide and crosslinking with ethylenediaminetetraacetic dianhydride (EDTAD) catalyzed by triethylamine. Oliveira et al. [21] synthesized cellulose acetate hydrogels at room temperature using 1,2,4,5-benzenotetracarboxylic dianhydride (PMDA) as a cross-linker. It is worth mentioning that the toxicity and degradation properties of the cross-linked molecules must be considered during the application of hydrogels prepared by chemical cross-linking methods, and non-biodegradable cross-linkers may be unfavorable in biological applications [22]. In addition, the hydrogels prepared by these two methods have no pore structure [23]. Extensive studies have illustrated that the pore structure is critical to the application of hydrogels [14,24]. For example, the pore structure of hydrogels in tissue engineering provides attachment points for cells, allowing cellular infiltration and nutrient exchange as well as the excretion of metabolic waste [25], allowing the storage and release of drugs in drug delivery [26,27]. Cellulose acetate hydrogels with non-porous structures undoubtedly limit the application range.

Cellulose acetate contains many unsubstituted hydroxyl groups, which are capable of originating hydrogen bonding-linked networks through dissolution regeneration. Among the many studies on cellulose acetate solubilized reproduction, the ternary system, consisting of cellulose acetate/acetone/anti-solvent, has been widely researched by scholars, such as the preparation of ultrafiltration membranes [28], electrospinning [29], and the research of the viscoelastic behavior of cellulose acetate in mixed solvents [30]. To our knowledge, there are no reports on the application of this ternary system for the preparation of hydrogels.

In this study, we prepared a cellulose acetate nanofiber hydrogel with a rich pore structure and good mechanical strength. The ternary system solution was prepared by introducing water molecules as an antisolvent into the cellulose acetate solution (dissolved in acetone) and strong anisotropic mechanical force was applied while introducing deionized water to prevent the local precipitation of cellulose acetate. Cellulose acetate molecules slowly agglomerate in the mixed solution due to the induction of water. This aggregation was further enhanced with the volatilization of acetone. Finally, cellulose acetate nanofiber hydrogel with pore structure was obtained via the exchange of acetone with water and the rearrangement of cellulose acetate in the exchange process. These hydrogels prepared at different rotational speeds were characterized to analyze the chemical properties, crystallinity variation, pore size distribution, specific surface area, thermal stability, and microstructure of cellulose acetate hydrogels. The mechanical properties of the composite hydrogels were investigated using compression tests. We believe that our work will broaden the application of cellulose acetate hydrogels in the biological field.

## 2. Results and Discussion

### 2.1. Scanning Electron Microscopy (SEM)

**Figure 1:**
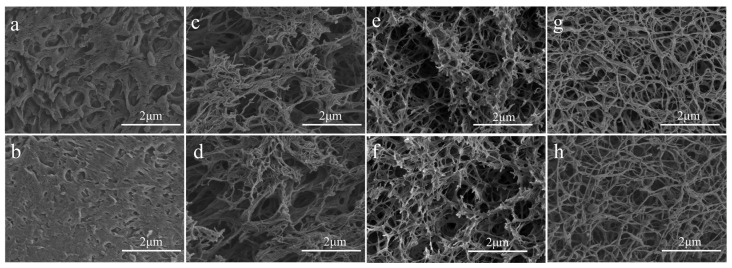
SEM micrographs of freeze-dried cellulose acetate (**a**,**b**) and cellulose acetate hydrogel prepared at different speeds. 1000 rpm (**c**,**d**), 1500 rpm (**e**,**f**), 2000 rpm (**g**,**h**).

Scanning electron microscopy (SEM) was used to observe the morphological structure of cellulose acetate fibers and cellulose acetate hydrogels prepared at different rotational speeds. Figure 1a,b shows the original structure of cellulose acetate. Figure 1c–h show the structures of cellulose acetate hydrogels prepared at 1000 rpm, 1500 rpm, and 2000 rpm, respectively. It can be seen that there is a significant difference between cellulose acetate and cellulose acetate hydrogel. The original cellulose acetate fibers exhibit a dense, non-porous bulk structure and the cellulose acetate hydrogel has a distinct three-dimensional network structure, rich pore structure with distinct fiber morphology, and a fiber diameter of about 30 nm. In addition, it can be observed that the fibrillar morphology of cellulose acetate hydrogels becomes more pronounced with increasing rotation speed, which may be due to the different degrees of deacetylation of cellulose acetate. According to the relevant literature [31], the network structure of hydrogels is produced via phase separation during the water substitution of acetone. The cellulose acetate hydrogels showed a distinct nanofibrous morphology, which may be due to the deacetylation of cellulose acetate during the dissolution and regeneration process [32].

### 2.2. Surface Area and Porosity Analysis

**Figure 2:**
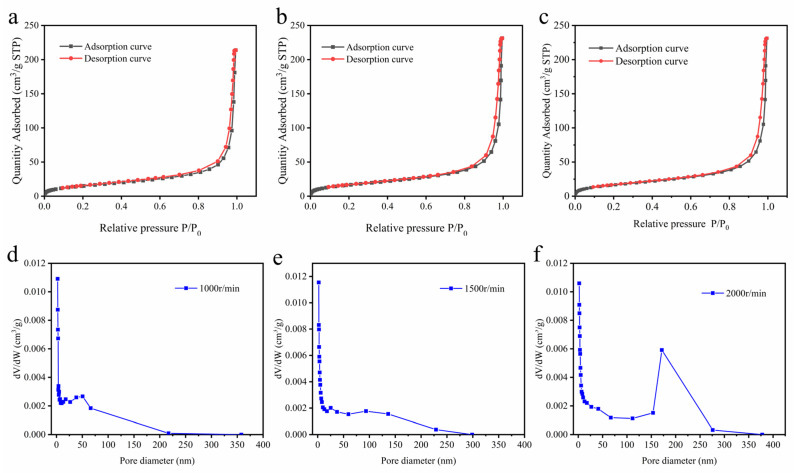
N_2_ adsorption (black line) and desorption (red line) isotherm (**a**–**c**) of CAH; (**a**) (1000 rpm), (**b**) (1500 rpm), (**c**) (2000 rpm). Pore size distribution of CAH. (**d**) (1000 rpm), (**e**) (1500 rpm), (**f**) (2000 rpm).

To further compare the differences in the pore structures of hydrogels prepared under different rotational speed conditions, the hydrogels were characterized by BET. Figure 2a–c shows the isotherms of N_2_ adsorption–desorption of cellulose acetate hydrogels prepared at 1000 rpm (a), 1500 rpm (b), and 2000 rpm (c), respectively. According to the classification of IUPAC, the cellulose acetate hydrogel was assigned as type II [33]. It can be observed that there is a hysteresis loop in the curve of cellulose acetate hydrogel in the high relative pressure range, which is due to the coalescence of mesoporous structures in the hydrogel, resulting in its desorption pressure being greater than the adsorption pressure [33]. The hysteresis loop between the adsorption and desorption curves indicated the presence of a mesoporous network in the gel [34]. Figure 2d–f shows the pore size distribution of cellulose acetate hydrogels prepared at 1000 rpm (d), 1500 rpm (e), and 2000 rpm (f), respectively. It can be seen that there are a large number of mesopores in the hydrogel, and the percentage of pore sizes of 150 nm to 250 nm in the hydrogel increases with the increase in the rotational speed. Table 1 shows that the pore size and specific surface area of the hydrogels increase with increasing rotational speed, which can be explained by the higher degree of deacetylation of cellulose acetate hydrogels under high rotational speed conditions. The pore structure of the hydrogel is significantly higher than that of the cellulose acetate hydrogel reported so far [21,35].

### 2.3. X-ray Diffraction (XRD) Analysis

**Figure 3:**
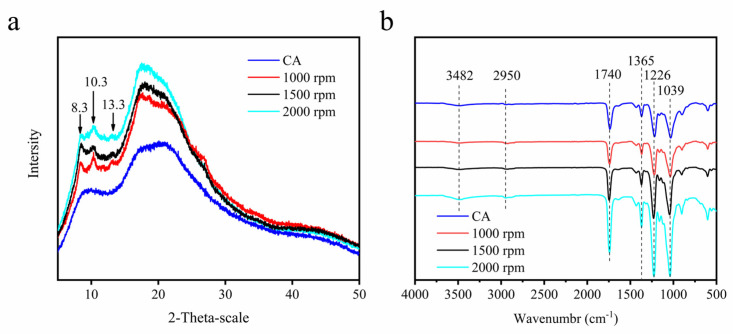
XRD patterns of cellulose acetate fibers and cellulose acetate hydrogel (**a**); FTIR spectra of cellulose acetate (CA) and cellulose acetate hydrogel (CAH) (**b**).

The characterization by XRD was performed to evaluate cellulose acetate hydrogels and cellulose acetate crystallinity. XRD diffraction curves are shown in Figure 3a, which show the typical morphology of semi-crystalline materials. The XRD diffraction curves of cellulose acetate show two broad “Mantle peaks” (i.e., peaks with weaker diffraction peak intensity), which is the result of introducing acetyl groups to broaden the interlayer space of the crystalline surface [36], indicating that the sample is a compound with a mixture of crystalline and non-crystalline structures and that the non-crystalline structure dominates [37]. The diffraction curves of cellulose acetate hydrogels are slightly different from those of cellulose acetate. Three sharp diffraction peaks exist in cellulose acetate hydrogels at 2θ = 8.3°, 10.3°, and 13.3°, which are usually considered the main features of cellulose semi-crystalline acetylated derivatives [38]. The broad diffraction peak around 2θ = 17.5° is attributed to the diffraction of the amorphous region of cellulose acetate hydrogels [34]. We used the Ruland–Vonk subtraction [39] (amorphous contribution subtraction method) to calculate the crystallinity index of cellulose acetate hydrogels and cellulose acetate. This method is based on the relationship between the area of crystalline domains by the total area. As shown in Equation (1) [31,40]. The CIs of cellulose acetate and cellulose acetate hydrogel were 9.6%, 10.3%, 11.7%, and 13.6%. Cellulose acetate hydrogels possess a higher crystalline phase than cellulose acetate fibrils. According to Freitas [34], the increase in crystallinity can be explained by the substitution of acetyl groups for hydroxyl groups that have a smaller volume than acetyl, which leads to larger organized chains and increased intermolecular interactions through hydrogen bonding. In summary, the degree of deacetylation of cellulose acetate in the water/acetone solvent mixture will be positively correlated with the intensity of mechanical action.

(1) $$\mathrm{CI}=\left(\frac{S_{c}}{S_{t}}\right)\times 100\%$$

where *S_c_* is the area of the crystalline domain and *S_t_* is the total area.

### 2.4. FTIR Analysis

The chemical structural characterization of cellulose acetate hydrogel was conducted by FTIR spectroscopy, as represented in Figure 3b, and characteristics of axial deformation vibration of bonds C–H aliphatic can be observed. The band appearing at 3470 cm^−1^ can be attributed to the stretching of hydroxyl groups [41]. A sharp band of cellulose acetate at 1741 cm^−1^ and the peak at 1365 cm^−1^ were attributed to carbonyl (C=O) group and (–OH) bending, respectively [40]; the peaks at 1741 and 1226 cm^−1^ belong to the carbon base and ester stretch, respectively; and the peak at 1039 cm^−1^ is the ether bond of the glycoside unit. This is consistent with that described in the literature [36]. The curves of cellulose acetate and cellulose acetate aerogel were similar and did not produce new characteristic peaks, which indicates that the hydrogel preparation process is a physical reaction of dissolution regeneration.

### 2.5. Thermal Properties of Cellulose Acetate and Cellulose Acetate Hydrogel

**Figure 4:**
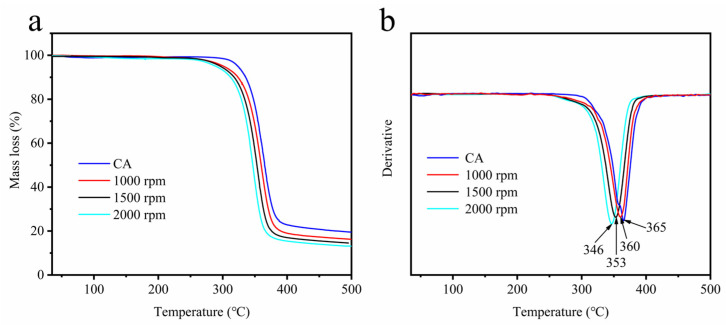
TG (**a**) and DTG (**b**) curve of CA and CAH.

The thermal stabilities of cellulose acetate and cellulose acetate hydrogel were examined using TGA. Figure 4a shows the TG curves of cellulose acetate and cellulose acetate hydrogel. Cellulose acetate and cellulose acetate hydrogel were degraded in three stages. There is a little mass loss before 100 °C, which is caused by the decomposition of water and volatile matter. Cellulose acetate degradation started at 300 °C and was completed at 400 °C with a sample loss of 77%, which is consistent with that described in the literature [20,39]. The degradation of cellulose acetate hydrogel started at 280 °C (1000 rpm), 274 °C (1500 rpm), and 268 °C (2000 rpm) with sample losses of 84%, 85.5%, and 87%. Figure 4b shows the DTG curves of cellulose acetate and cellulose acetate hydrogel. Cellulose acetate degraded fastest at 365 °C. The TG and DTG curves show that the hydrogel is less thermally stable than the cellulose acetate and the fastest thermal degradation temperature of cellulose acetate hydrogels decreased with increasing rotational speed. Chen et al. [41] and Peter et al. [42] investigated the thermal stability of cellulose acetate and indicated that the thermal stability of cellulose acetate increased with increasing DS, which was attributed to the formation of a new ordered structure of cellulose acetate in the substitution region. The increase in rotational speed intensified the degree of the deacetylation of cellulose acetate and disrupted the ordered structure of the substitution region, thus reducing the thermal stability of cellulose acetate hydrogels [38].

### 2.6. Compressive Strength of Hydrogel Scaffolds

**Figure 5:**
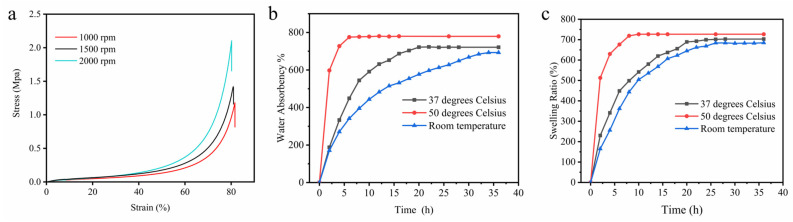
Compressive stress–strain curves of the CAH (**a**); water absorbency curves (**b**), and swelling curves (**c**) of CAH at various temperatures.

Compression tests were performed to evaluate the mechanical strength of the cellulose acetate hydrogels prepared at different rotational speeds, as shown in Figure 5a. All samples support a slight increase in load at the beginning of the test, followed by a sharp increase in load at strains above 60%. When the deformation reached 80%, the compressive strength of the hydrogel could reach up to 2.1 MPa (2000 rpm), 1.4 MPa (1500 rpm), and 1.2 MPa (1000 rpm), respectively. At this time, the hydrogel underwent irreversible plastic deformation, and there was no obvious rupture of the hydrogel after plastic deformation. The compression tests of the hydrogels were similar to those reported in the literature [41]. The improvement of the mechanical properties of the cellulose acetate hydrogels with the increase in the rotational speed can be explained by the deacetylation reaction of the cellulose acetate hydrogels increasing their crystallinity, and the increase in crystallinity makes the structure of cellulose acetate more orderly, and thus improves the mechanical properties of cellulose acetate.

### 2.7. Water Absorption and Swelling of Hydrogel Scaffolds

Combining the above characterization, hydrogel prepared at 2000 rpm has the best mechanical properties, specific surface area, and pore size. To evaluate the potential of cellulose acetate hydrogels for practical applications, swelling and water absorption tests at various temperatures were performed on the hydrogels prepared at 2000 rpm. Figure 5b shows the water absorption curve of cellulose acetate hydrogel. The water absorption of hydrogel increases slightly with increasing temperature, and the water absorption rates were 691%, 721%, and 778% at room temperature, 37 °C, and 50 °C, respectively. The time to reach water absorption equilibrium decreases with the increase in temperature, which was 6 h, 18 h, and 32 h, respectively. The water absorption of hydrogels is significantly higher than that of cellulose derivatives [23]. The hydrogel exhibited high water absorption, indicating that the hydrogel was a highly absorbent hydrogel. Figure 5c shows the swelling behavior of cellulose acetate hydrogel in PBS buffer solution at different temperatures. The swelling rate of the hydrogel increased rapidly in the first few hours, and the time to reach the swelling equilibrium decreased with the increase in temperature, which was 8 h, 22 h, and 26 h, respectively. All the hydrogels immersed in water and PBS solution kept the same shape and did not break, indicating that the hydrogels have good stability. Hydrogels with high water absorption and swelling stability are very important for their wide application in the biomedical field. The percentage of swelling ratio (SR) and water absorption was calculated according to the following equation [17]:

(2) $$\mathrm{SR}\;(\%)=\frac{w_{s}-w_{0}}{w_{0}}\times 100\%$$

where $w_{0}$ is the weight of the freeze-dried hydrogel and $w_{s}$ is the weight of swollen hydrogels.

## 3. Conclusions

In this study, nano-cellulose acetate hydrogels with rich pore structures were prepared using the physical cross-linking method. The introduction of water into cellulose acetate solution as an antisolvent induces the limited phase separation of cellulose acetate, and, finally, hydrogels are obtained via the water replacement of acetone. SEM characterization results show that the hydrogel possesses a large number of pore structures and obvious nanofiber morphology, and the fiber morphology becomes more and more obvious with the increase in rotational speed. The BET test results showed that the average pore size of the hydrogel prepared at a rotational speed of 2000 rpm was 26 nm and the specific surface area was 62 m^2^/g. This method can be extended to the preparation of hydrogels from polymers with phase separation properties. Cellulose acetate is derived from cellulose and has the advantages of being renewable and sustainable. Cellulose acetate hydrogels have potential applications in the field of tissue engineering scaffolds and cell culture carriers.

## 4. Materials and Methods

### 4.1. Materials

Cellulose acetate (DS = 2.5) was provided by Macklin Biochemical Technology Co., Ltd. (Shanghai, China). Acetone was supplied by Nanjing Chemical Reagent Co., Ltd. (Nanjing, China). Phosphate-buffered saline was supplied by Cytiva. All the chemicals used in the study were of analytical grade and without further refinement. All experiments used deionized water.

### 4.2. Preparation of Cellulose Acetate Hydrogel

**Figure 6:**
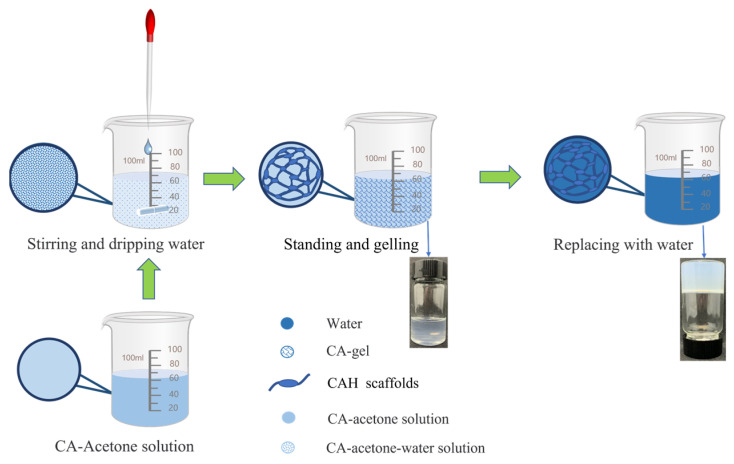
Preparation process of cellulose acetate hydrogel (CAH).

The preparation of cellulose acetate is shown in Figure 6. Acetone (20 g) was added to the beaker, and then 4 g of cellulose acetate (DS = 2.5) was added to the acetone and stirred airtight for 2 h at room temperature with a magnetic stirrer at 300 rpm. As a result, a clear solution was obtained, indicating that the cellulose acetate was well dissolved. Then, acetone and deionized water were mixed at a ratio of 1:1. In the next step, 20 g of water–acetone solution was slowly added to the reaction system using a pipette under stirring at 2000 rpm. The mass ratio of cellulose acetate, water, and acetone in the reaction system was 2:5:8 by volatilizing the acetone. The resulting ternary system was poured into test tubes and left sealed at room temperature for 24 h until a gel was formed. The gel was immersed in water for 48 h. The gel was converted into hydrogel by solvent exchange, and the water was changed every 8 h.

### 4.3. Scanning Electron Microscopy (SEM) Analysis

The microstructure of cellulose acetate hydrogel was studied using a JSM-7600F pasted electron microscope (Japanese Institute of Electronics, Tokyo, Japan). The samples were fixed in a double-sided adhesive tape mounted on an aluminum specimen stub and coated with a thin layer of gold before microscopic examination [42].

### 4.4. Specific Surface Area and Pore Size Distribution of Cellulose Acetate Hydrogel

The specific surface area and pore size distribution of the samples were measured using the Brunauer–Emitt–Teller (BET) method of a fully automated specific surface area and pore analyzer (Quadra V-Sorb 2800P, Wuhan, China). Each sample was freeze-dried and degassed at 130 °C for 6 h prior to testing. N_2_ was used as the adsorbent for the tests at 77 K.

### 4.5. XRD Analysis

The crystallinity of the cellulose acetate and cellulose acetate hydrogel was measured on an XRD instrument (XRD, Ultima IV, Rigaku, Japan) with a scattering angle from 5° to 50° at a scanning speed of 2°/min.

### 4.6. Attenuated Total Reflectance Fourier-Transform Infrared (ATR-FTIR) Spectroscopy

The chemical structures of the dried hydrogels were characterized via a Fourier transform infrared spectroscope (VERTEX 80 V, Bruck, Germany), and the wavenumbers ranged from 500 to 4000 cm^−1^.

### 4.7. Thermogravimetric Analysis (TGA)

Thermal gravimetric analysis (TGA) of CA and the freeze-dried CAH was performed using an SDT Q600 device (Mettler Toledo, A39, Zurich, Switzerland). The samples were analyzed at temperatures ranging from 30 to 500 °C with a heating rate of 10 °C/min and a nitrogen flow rate of 20 mL/min.

### 4.8. Mechanical Properties

The hydrogel samples were soaked in distilled water to achieve equilibrium expansion before conducting the tests and were then cut into cylindrical specimens 24 mm in diameter. Compression tests were performed on a universal testing machine (Q500 from TA Company, New Castle, DE, USA) at room temperature, and then the stress–strain curves were recorded for each sample. In mechanics performance testing, three consecutive experiments were performed and the data were expressed as mean.

### 4.9. Water Absorption, Swelling Studies of Hydrogel Scaffolds

For the swelling characterization, the freeze-dried hydrogels were weighed and immersed in phosphate-buffered saline (PBS, pH 7.4) and deionized water at various temperatures. The swollen hydrogels were removed from the buffer at different time intervals and weighed after removing surface water with filter paper.

## Figures and Tables

**Table 1:** The pore and specific surface area sizes of hydrogels prepared at different rotational speeds.

Sample (rpm)	Diameter (nm)	Specific Surface Area (m^2^/g)
1000	23.078 ± 1.121	56.035 ± 0.753
1500	24.651 ± 1.312	58.281 ± 0.647
2000	26.594 ± 1.034	62.021 ± 0.712

## Data Availability

Data are available from the authors. Samples of the compounds are available from the authors.
